# Analyzing Predictive Factors for the Media’s Impact on the Nursing Profession

**DOI:** 10.3390/nursrep15010025

**Published:** 2025-01-16

**Authors:** Jadranka Pavić, Marta Marković, Ana Marija Hošnjak, Aleksandar Racz, Irena Kovačević, Martina Smrekar

**Affiliations:** 1 Department of Nursing, University of Applied Health Sciences, 10000 Zagreb, Croatia; jadranka.pavic@zvu.hr (J.P.); aleksandar.racz@zvu.hr (A.R.); irena.kovacevic@zvu.hr (I.K.); martina.smrekar@zvu.hr (M.S.); 2 Department of Nursing, Faculty of Health Studies, University of Rijeka, 51000 Rijeka, Croatia; 3 Sestre Milosrdnice University Hospital Center, 10000 Zagreb, Croatia; martamarkovic2602@gmail.com; 4 School of Medicine, University of Rijeka, 51000 Rijeka, Croatia

**Keywords:** media, nurses, education

## Abstract

**Introduction:** Mass media play a crucial role not only in informing the public but also in shaping public perception, educating, and enhancing the visibility of various professions, including nursing. Despite being the most populous healthcare profession, nursing remains underrepresented in media coverage. This imbalance affects the social status of the nursing profession and its public perception. **Methods:** This cross-sectional study utilized a validated questionnaire with high internal reliability (Cronbach’s alpha coefficients) to assess nurses’ perceptions of the media’s role in society and the nursing profession. Data were collected from 203 participants using an online survey employing the snowball sampling method. Statistical analyses included Welch ANOVA, *t*-tests, and hierarchical regression to predict the importance of media education. **Results:** Participants demonstrated positive perceptions of the media’s societal influence but identified a lack of adequate representation of nurses. Younger nurses and those with higher education levels emphasized the need for media education. Regression analysis revealed that perceptions of the media’s power and self-assessed media competencies were significant predictors of valuing media education. **Conclusions:** The findings highlight the need for integrating media literacy training into nursing education to enhance professional visibility and public engagement. This can empower nurses to actively contribute to shaping their professional image and addressing public misconceptions. Future research should expand the sample size and explore diverse healthcare settings to validate these findings.

## 1. Introduction

The reputation of a profession is shaped by societal perceptions, which encompass socioeconomic status, opportunities for individual professional growth, and the profession’s global development and influence within the dynamics of societal events [1]. Given that the media influence the reputation of the profession directly and indirectly, they have a strong influence on the reputation of nursing as well. The influence of the media on nursing is reflected through an indifferent relationship with nursing on the one hand or through the influence of creating prejudice and stereotypes towards nursing on the other hand [2].

Why is nursing not present in the media? It is because nurses and their organizations have failed to recognize the importance of the media and communicate with the media adequately. Furthermore, the media are also one of the reasons, with their focus on profit, which is related to sensationalism, selling content, and a lack of knowledge about nursing, which is often the reason for prejudice and the stigmatization of nursing in the media. An overview of historical representations of nurses in the media shows that nursing has always been associated with stereotypes, which have changed over time [3]. In TV shows, nurses are always depicted as inferior in the healthcare team, subordinated to doctors, and lacking competent skills [4].

The Woodhull study examined media coverage of nursing, highlighting the limited visibility and representation of nurses in health-related news. This study was initiated by the international nursing society Sigma Theta Tau in 1998 and was designed to document attitudes about nurses and the nursing profession in the media, stating that nurses are the largest and most prominent demographic group within healthcare, they are on the first line and providing the most hands-on interaction between providers and patients. For that reason, nursing should be covered in the media. Research on a sample of 2000 analyzed articles on healthcare revealed that nurses were cited only 4% of the time and that this citation was superficial [5]. This study was repeated twenty years later (2018) under the title The Woodhull Study Revisited, but the number of articles mentioning nurses has not statistically significantly changed in 20 years [6]. This study initiated a comprehensive discussion on the importance of dialogue between nurses and journalists, meeting and discovering possibilities in mutual interaction to develop more efficient communication channels. This is important in enabling a comprehensive depiction of health and disease as well as the role of nurses in healthcare and society as a whole [6]. Over time, certain improvements can be observed regarding the perception of nurses by the media. The increase in resources which are invested in nursing resulted in an increase in the education degree and expertise in the nursing profession. According to the authors, for the negative effects of nursing to disappear, nurses should take measures to protect the nursing profession from misrepresentation and support nurses who maximize the positive effect that YouTube as a promotional tool can have [7]. Furthermore, the research on 1261 relevant studies searched in Medline, Cinahl, and PsychINFO databases in the period between 1997 and 2010 showed that the actual image of nursing in the public is diverse and unharmonized. According to the authors, this image is partially created by the nurses themselves due to their invisibility and lack of public discourse. For that reason, nurses and medical technicians should increase their presence in the media to improve the general population’s perception of nursing and to take a stronger position in medical organizations [8].

Although there has been a slight shift in the media’s perception of nursing in the last ten years, it was still minimal. However, with the outbreak of the COVID-19 virus pandemic, it has become clear to the public that the coronavirus crisis has shown the great professional contribution of nurses to the suppression and prevention of the epidemic and the treatment and health care of the sick. In a very short time period, and without the necessary experience, in a completely unknown and uncertain situation, nurses participated in the reorganization of departments and adapted their work to specific circumstances. These activities were noticed by patients, families, and colleagues in the team, and, during the pandemic, nurses became the focus of media and public interest [9].

From this experience, the importance of covering the work of nurses in the media is important. Nurses can change the perception of the nursing profession in the public through the education of the media, increasing their knowledge of the influence and power of the media in society, and increasing their personal competencies for participating in the media. This is important because of the contribution to the media literacy of the population through inclusion in health education through the media, as well as the contribution to the status and reputation of the nursing profession in society.

The purpose of this research is to assess nurses’ perceptions of the media’s role in the nursing profession, focusing on how these perceptions vary based on their experience and qualifications. It also aims to explore how media education for nurses is valued and what factors predict its importance.

For this reason, the main goal of this research was to assess nurses’ perception of the importance of media for the nursing profession through the following areas:

- To examine the differences in the perception of power and the role of the media in society based on nurses’ length of service and qualifications.
- To examine the differences in the perception of nurses in the media based on their length of service and qualifications.
- To examine the differences in the perception of the importance of educating nurses on the media based on their length of service and qualifications.
- To examine the differences in the perception of personal competencies in the media based on length of service and qualifications.
- To examine the predictive value of the variables of the perception of power and the role of the media in society, the perception of a nurse in the media, and the assessment of personal competencies in the media in explaining the perception of the importance of educating nurses on the media.

## 2. Materials and Methods

The research was approved by the Committee for Professional and Scientific Research of the University of Applied Health Sciences Zagreb, reference number 251-379-1-20-02, as of 1 July 2020. Participants provided informed consent electronically, confirming their understanding of this study’s purpose and voluntary participation.

A questionnaire with four scales was created for this research. A snowball sampling method was employed for its ability to reach diverse participants through peer referrals. This method facilitates recruitment in settings where direct access to participants is limited but may restrict generalizability. The sample size of 203 was chosen based on prior studies and statistical power considerations. Data were collected using an online survey to ensure anonymity and convenience, though this method may introduce selection bias.

The data were analyzed using IBM SPSS Statistics (Version 26).

Before the statistical analyses, the assumptions for their implementation were checked. All variables had indices of asymmetry and flatness within acceptable limits, so the data were processed using parametric methods.

Because the condition of homogeneity of variance was not met for any variable, Welch ANOVA was used to test differences in the examined variables between subjects of different lengths of service, and, for subsequent comparisons between groups, the Games–Howell test was used. Differences in the investigated variables between subjects with college/university and secondary education were tested with a *t*-test. To examine the predictive value of the variables of perception of the power and role of the media in society, perception of nurses in the media, and assessment of personal competencies in the media to explain the perception of the importance of education of nurses in the media, a hierarchical regression analysis was used in which the first step was to control professional education and years of experience.

### 2.1. The Scale of Perception of the Power and Role of the Media in Society

This scale was developed based on frameworks proposed by García and Qureshi [2]. It measures perceptions of media power and role in society, with high internal consistency (Cronbach’s alpha = 0.965), and explains 91.65% of the total variance.

This scale was developed for the needs of this research, and it measures the perception of the power and role of the media in society. It consists of eight statements (for example, *The media can significantly influence people’s attitudes*). The respondents assess the statements on a 5-degree scale (1—I completely disagree; 5—I fully agree).

The score on the scale is the average assessment of the participants on all statements, and a higher score indicates a more pronounced perception of the power and role of the media in society.

### 2.2. Nurse Perception Scale in the Media

This scale was adapted from the work of Hoeve et al. [8]. It measures perceptions of nurses’ representation in the media. It showed good reliability (Cronbach’s alpha = 0.804) and explained 63.9% of the variance.

This scale was developed for the needs of this research, and it measures the perception of nurses in the media. It consists of seven statements (for example, *Nurses are under-represented in the media*). The respondents assess the statements on a 5-degree scale (1—I completely disagree; 5—I fully agree).

The score on the scale is the average assessment of the participants on all statements, and a higher score indicates a more favorable perception of nurses in the media.

### 2.3. The Scale of Perception of the Importance of Educating Nurses on the Media

This scale was inspired by research conducted by Mason et al. [6]. It measures the perceived need for media education among nurses. Reliability analysis showed Cronbach’s alpha = 0.804 with 63.9% variance explained.

This scale was developed for the needs of this research, and it measures the importance of educating nurses on the media. It consists of seven statements (for example, *Nurses need more education on media usage*). The respondents assess the statements on a 5-degree scale (1—I completely disagree; 5—I fully agree).

The score on the scale is the average assessment of the participants on all statements, and a higher score indicates a bigger perception of the importance of educating nurses on the media.

### 2.4. Scale of Assessment of Personal Competencies in the Media

This scale was developed based on the work of Kemmer and Silva [10]. It measures nurses’ self-assessed media competencies.

This scale was developed for the needs of this research, and it measures the assessment of participants about personal competencies in the media. It consists of eight statements (for example, *I believe that I can be a competent interlocutor about healthcare in television shows*). The respondents assess the statements on a 5-degree scale (1—I completely disagree; 5—I fully agree).

Exploratory factor analysis performed using the method of principal components resulted in two factors which explained 85.45% of the total variance. Given that the second factor relates to three negatively formulated statements and those before the rotation have high loadings, we concluded that it is a single factor. The internal reliability coefficient Cronbach alpha is 0.672.

The score on the scale is the average assessment of the participants on all statements, and a higher score indicates a higher assessment of personal competence in the media.

## 3. Results

### 3.1. Descriptive Factors for the Examined Variables

From Table 1, it is evident that the participants estimate the power and role of the media in society and the importance of educating on the media as slightly above average, while their competencies in the media are assessed as average, and the perception of nurses in the media remains slightly below average. Data collection occurred during March 2022, a period influenced by heightened public awareness of nursing due to the COVID-19 pandemic.

All variables showed high or moderately high positive correlations, with the highest correlation recorded between the variables *Strength and the role of media in society* and *Assessment of the importance of media education* (r = 0.86, *p* < 0.01). The only statistically insignificant correlation was between the perception of nurses in the media and the assessment of personal media competencies (r = 0.13, *p* > 0.05).

The mean values indicate that participants rated The power and role of the media in society the highest (M = 3.51), followed by The assessment of the importance of educating on the media (M = 3.42), with the lowest rating for The perception of nurses in the media (M = 2.57), while the standard deviations show the greatest variability in responses for The perception of nurses in the media (SD = 1.93) and the least for The assessment of the importance of educating on the media (SD = 1.24), reflecting more consistent responses in the latter.

### 3.2. Differences in Examined Variables Based on Length of Service

The Welch ANOVA results (see Table 2) showed that participants with different lengths of service differ significantly in the assessment of the power and role of the media in society. The participants with a length of service of 21–30 years assess the role and power of the media in the society significantly lower than all other participants, who assess the power and role of the media in the society above average.

Additionally, the differences between the groups for each examined variable are further outlined in Table 3, which shows the statistical significance of these differences based on length of service.

### 3.3. Differences in Examined Variables Based on Education

The participants are divided into two categories based on education (see Table 4). The participants with college and university education were merged into one category because there were few participants with university education. The other category consisted of participants with high school education. The differences between those two groups were significant for all examined variables. Participants with university/college education expressed higher assessments of the role and power of the media in society, a better perception of nurses in the media, a higher perception of the importance of education in the media, and a better assessment of personal competencies in the media.

### 3.4. The Predictors of the Assessment of the Importance of Educating on the Media

To check to what extent the variables of perception of the power and role of the media in society, perceptions of nurses in the media, and assessments of personal competencies in the media explain the perception of the importance of the education of nurses in the media, a hierarchical regression analysis was conducted. In the first step, education and length of service were included as control variables, and, in the second one, the dependent variables were included as control variables.

In the first step of the analysis, 42% of the total variance of the criterion variable was explained, and this was statistically significant (R^2^ = 0.42, *p* < 0.000). Significant predictors were professional education and work experience. At the same time, the participants with university education compared to those with college education gave higher assessments of the importance of educating on the media extent (β = 0.28 ***). Furthermore, the participants with longer working experience compared to those with shorter working experience gave lower assessments of the importance of educating on the media (β = −0.53 ***). Therefore, younger participants and participants with university education are more aware of the importance of educating on the media (Table 5). In the second step, after introducing the remaining predictor variables (The *role and power of the media*, *The perception of nurses*, *Personal competencies*) into the analysis, a total of 77% of the variance of the predictor assessment of the importance of educating on the media variable was explained (R^2^ = 0.42, *p* < 0.000).

In the second step of the analysis (after the control of education and length of service), 35% of the total variance of the criterion variable was additionally explained, and this was significant. Significant predictors were the perception of the role and power of the media in society (β = −0.70 ***) and the assessment of personal competencies in the media (β = −0.53 **) (Table 5). Therefore, the participants who give more importance to the role and power of the media in society and have personal competencies for the media are more aware of the importance of educating on the media.

## 4. Discussion

This study adds to the literature by highlighting the predictive value of perceptions of media power and self-assessed competencies in valuing media education. The results emphasize the need for incorporating media literacy into nursing education to address the profession’s underrepresentation in media. Practical solutions include organizing workshops and establishing partnerships with journalists to foster positive representations of nursing.

Numerous studies confirm the awareness of nurses regarding the importance of media in society and its influence on the public’s perception of health information and the profession’s image. For example, a study on the attitudes of nurses toward the impact of mass media on the public perception of nursing and user interactions conducted in the United Kingdom showed that nurses are aware of the media’s significance in society and its impact on healthcare service users. In this research, nurses believe that the media can significantly influence the public, acting as intermediaries that may lead to the erosion of professional boundaries, posing a challenge to professional authority and autonomy [11].

Furthermore, in a study conducted among nurses in primary health care, whose primary role is educating the population, results indicated that nurses reported that their patients were often influenced by controversial health stories published in the media. This influence affected patients’ perceptions and decisions about care, consequently increasing the workload for nurses. The strong impact of the media required additional efforts from nurses to educate patients about information or incomplete information from the media [12].

Participants in our study assess the perception of nurses in the media as below average. Similar results are shown in other studies conducted among nurses, such as that of Lupieri and Kičić [13] in which 84% of respondents believed that nurses were not adequately represented in the media. In that study, 81% of participants stated that nursing is not a popularized profession in the media, and 86% mentioned being bothered by the influence of prejudices on the portrayal of nurses in the media. Furthermore, research analyzing the portrayal of nursing in television series and movies indicates that nurses are often marginalized and depicted performing extremely simple tasks such as transporting patients, changing bed linens, measuring blood pressure, or engaging in romantic relationships with doctors [14,15,16].

Media experts also confirm the lack of inclusion of nurses in media content due to a lack of understanding of the nursing profession’s scope of work and the invisibility of nurses in society. In Kemmer and da Silva’s study [10] on journalists’ attitudes toward the visibility of the nursing profession in the media, the responsibility and passivity of nurses in engaging with the world of the media were emphasized. Participants in this study highlighted the need to build a more coherent image of nursing and nurses by exposing the profession primarily to the media to reach the wider population [10].

Despite awareness of the power and influence of the media on society, our study showed that nurses with from 21 to 30 years of work experience perceive significantly lower power and role of the media in society compared to all other participants (Table 2). These differences can be attributed to a generational perspective, wherein older generations belonging to Generation X attach significantly less importance to mass media than Generation Y, often described as digital natives, who grew up in the era of media dominance [17,18].

Furthermore, research on nursing students’ attitudes toward the use of social media in shaping the public image of nurses shows that younger generations, future nurses, are fully aware of the strong influence of the media on the public and thus on shaping the image of the nursing profession in society [19].

Furthermore, participants with university/college education expressed higher assessments of the role and power of the media in society, a better perception of nurses in the media, a higher perception of the importance of education in the media, and a better assessment of personal competencies in the media compared to participants with high school education (Table 3). The education of nurses is crucial for the development of both competencies and professionalization. Research confirms that there are differences in the attitudes of nurses and technicians about the nursing profession and education about their level of professional education and self-assessment of competence, whereby nurses with a lower level of education show lower results [20,21].

The effects of education generally have a positive impact on changes in the economic, social, sociocultural, and natural environment through a breadth of information that influences the affective level, including attitudes and values [22]. Since it is important to raise awareness of the importance of education on the media, which today, in the globalized world, are leading in providing information as well as determining the image of persons or professions and public opinion, we examined the predictive value of the variables of the perception of the power and role of the media in society, the perception of nurses in the media, and the assessment of personal competency in the media in explaining the perception of the importance of educating nurses on the media. Participants with a university education compared to those with a college education gave higher estimates of the importance of media education, while participants with a longer working experience compared to those with a shorter working experience gave lower estimates of the importance of educating on the media. These control variables explained 42% of the total variance of the criterion variable, i.e., the importance of educating nurses on the media, while, in the second step of the hierarchical regression analysis, 35% of the total variance of the criterion variable about the perception of the importance of educating nurses on the media was explained. This analysis confirmed that the participants who give more importance to the role and power of the media in society and have personal competencies for the media are more aware of the importance of education on the media.

Research conducted by van Bekun and Hilton [12] indicates that nurses recognize the need to enhance their media literacy skills to develop critical autonomy regarding all media. This enhancement allows for more meaningful conversations with patients about their health issues and choices, enabling nurses to actively engage in the media. Additionally, research by Hoyle et al. [11], based on the findings about media influence and the need for media education, concludes that a closer collaboration between nurses and the media is necessary. This collaboration should encourage the development of critical inquiry skills focused on media during nursing education, involving continuous practical engagement and achieving a shared understanding of both the media and the nursing profession.

This study emphasizes practical implications for nursing education and professional practice. Integrating media literacy into nursing education can empower professionals to effectively address media misconceptions, enhance the profession’s public image, and strengthen advocacy for healthcare policies.

Further research is needed to explore the evolving role of media in nursing practice across different healthcare settings and cultural contexts. Longitudinal studies could provide insights into how nurses’ media engagement changes over time, while qualitative research may uncover the nuanced ways in which media influences clinical decision-making and patient interactions. Additionally, investigating the impact of specific media types on nursing outcomes and professional development could enhance our understanding of effective media utilization in the field.

The limitations of this study are as follows: it was conducted in a single healthcare institution due to logistical feasibility; therefore, this study’s generalizability is inherently limited. Future research should aim to include participants from diverse institutions and regions, enabling broader insights into the media’s role in nursing.

## 5. Conclusions

This research showed that there are differences in the perception of the power and role of the media in society depending on the length of service and education of nurses.

Nurses with longer working experience and older age rates significantly lower the importance of the media, i.e., their strength and role in society than all other participants, while nurses with a higher or higher professional education state a higher level of power and role of the media in society.

In addition, nurses with university or college education show higher values in assessing the perception of nurses in the media and the importance of educating nurses on the media, and they have a higher assessment of personal competencies in the media.

However, nurses with a university education, compared to the ones with a college education, assessed the value of the importance of educating nurses on the media on a higher level, which shows that, the higher the education level, the greater the awareness of the importance of education. In addition, nurses who give more importance to the power and role of the media than other respondents and perceive a higher level of personal competence for the media also show a higher level of awareness of the importance of educating nurses on the media. These results emphasize the importance of education and continuous training for nurses in media literacy and the acquisition of media competencies.

## Figures and Tables

**Table 1:** Basic descriptive parameters and correlations between examined variables.

	1	2	3	4
1. The power and role of the media in the society	-	0.58 **	0.86 **	0.34 **
2. The perception of nurses in the media		-	0.52 **	13 **
3. The assessment of the importance of educating on the media			-	0.44 **
4. The assessment of personal competencies in the media				-
M	3.51	2.57	3.42	2.96
SD	1.53	1.93	1.24	1.79
Range of results	1–5	1.14–5	1.43–5	1–5
Asymmetry index	−0.853	0.523	−0.490	0.346
Flatness index	−0.939	−0.795	−1.278	0.315

** *p* < 0.01, the correlation is significant at the 0.01 level (two-tailed).

**Table 2:** Descriptive factors for the examined variables based on length of service.

	The Power and Role of the Media		The Perception of Nurses in the Media		The Importance of Educating on the Media		Personal Competencies in the Media	
	M	SD	M	SD	M	SD	M	SD
0–4	4.45	0.39	3.15	90 **	4.19	0.59	3.07	1.04
5–10	4.22	0.63	2.92	87 **	3.88	0.78	2.98	90 **
11–20	4.39	0.61	2.88	72 **	3.91	0.75	3.22	81 **
21–30	1.84	1.46	1.81	58 **	2.23	1.17	2.68	52 **
above 30	4.28	0.82	2.79	80 **	4.02	0.99	3.17	57 **

** *p* < 0.01, the correlation is significant at the 0.01 level (two-tailed).

**Table 3:** Differences in examined variables based on length of service.

	F	*p*	Difference Between Groups
1. The power and role of the media	73.13	*p* < 0.001	1-4. 2-4. 3-4. 5-4
2. The perception of nurses in the media	26.752	*p* < 0.001	1-4. 2-4. 3-4. 5-4
3. The importance of educating on the media	41.691	*p* < 0.001	1-4. 2-4. 3-4. 5-4
4. Personal competencies in the media	3.748	*p* < 0.001	3-4. 5-4

**Table 4:** Differences in examined variables based on education.

	The Power and Role of the Media		The Perception of Nurses in the Media		The Importance of Educating on the Media		Personal Competencies in the Media	
	M	SD	M	SD	M	SD	M	SD
College and university	4.47	0.48	2.84	0.91	4.19	0.66	3.27	88 **
High school	2.69	1.66	2.34	0.89	2.73	1.22	2.69	58 **
*t*-test	10.528 ***		3.959 ***		10.768 ***		5.613 ***	

** *p* < 0.01, the correlation is significant at the 0.01 level (two-tailed). *** *p* < 0.001, the correlation is statistically significant at the 0.001 level (two-tailed)

**Table 5:** The predictors of the assessment of the importance of educating on the media.

	Step 1 (β)	Step 2 (β)
Education	0.28 ***	0.11 **
Length of service	−0.53 **	−0.03
The role and power of the media		0.70 ***
The perception of nurses		0.05 **
Personal competencies		0.15 **
R^2^	0.42	0.77
ΔR^2^		0.35
F	73.17 ***	99.41 ***

*** *p* < 0.001; ** *p* < 0.01.

## Data Availability

Data is contained within the article.

## Public Involvement Statement

No public involvement in any aspect of this research.

## Guidelines and Standards Statement

This manuscript was drafted against the STROBE checklist for cross-sectional research.
